# A Review of Completed and Ongoing Complement Inhibitor Trials for Geographic Atrophy Secondary to Age-Related Macular Degeneration

**DOI:** 10.3390/jcm10122580

**Published:** 2021-06-11

**Authors:** Omar A. Halawa, Jonathan B. Lin, Joan W. Miller, Demetrios G. Vavvas

**Affiliations:** Department of Ophthalmology, Massachusetts Eye and Ear and Harvard Medical School, Boston, MA 02115, USA; Omar_Halawa@hms.harvard.edu (O.A.H.); jblin6@gmail.com (J.B.L.)

**Keywords:** complement inhibitors, age-related macular degeneration, AMD, exudation, exudative AMD, wet AMD, C3, C5, Factor D

## Abstract

Age-related macular degeneration (AMD) is a leading cause of irreversible blindness among older adults in the Western world. While therapies exist for patients with exudative AMD, there are currently no approved therapies for non-exudative AMD and its advanced form of geographic atrophy (GA). The discovery of genetic variants in complement protein loci with increased susceptibility to AMD has led to the investigation of the role of complement inhibition in AMD with a focus on GA. Here, we review completed and ongoing clinical trials evaluating the safety and efficacy of these studies. Overall, complement inhibition in GA has yielded mixed results. The inhibition of complement factor D has failed pivotal phase 3 trials. Studies of C3 and C5 inhibition meeting their primary endpoint are limited by high rates of discontinuation and withdrawal in the treatment arm and higher risks of conversion to exudative AMD. Studies evaluating other complement members (CFB, CFH, CFI and inhibitors of membrane attack complex—CD59) are ongoing and could offer other viable strategies.

## 1. Introduction

**Figure 1:**
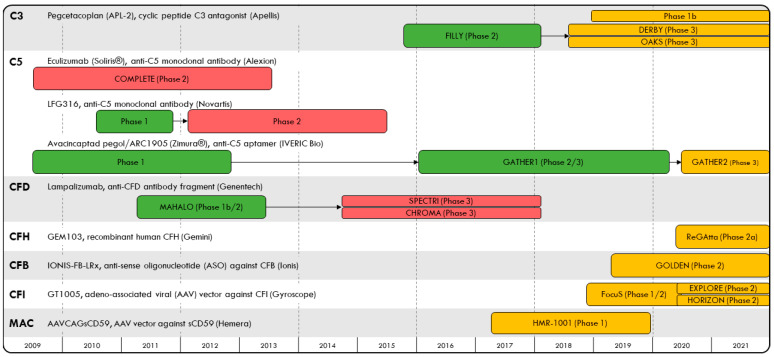
Timeline of complement inhibitor trials evaluating safety and efficacy in geographic atrophy to-date, organized by their complement protein targets.

Age-related macular degeneration (AMD) has an estimated global prevalence of 8% and is a leading cause of blindness among older adults [1]. Exudative AMD (eAMD) is characterized by abnormal blood vessel growth and choroidal neovascularization and has been treated with anti-vascular endothelial growth factor (VEGF) therapies administered by intravitreal injection. Advanced, non-exudative AMD results in geographic atrophy (GA), defined as degeneration of photoreceptors, retinal pigment epithelium cells and choriocapillaris. There are currently no approved therapies shown to prevent progression to GA among non-exudative AMD patients. At the turn of the 21st century, several landmark genome-wide association studies discovered complement factor gene variants that are associated with susceptibility to AMD, including complement factors H, I, B, D and 2, among others [2,3,4,5,6,7]. While dysregulation of the complement system, an essential component of the innate immune system, is thought to be associated with an increased risk for AMD, its role in AMD pathogenesis remains unclear; genetic variants found to predict susceptibility to AMD have not been associated with GA progression [8,9], and in some studies, C3 risk alleles are actually associated with decreased progression of GA [10]. Nevertheless, given the potential relationship between complement dysregulation and the risk of GA secondary to AMD, several studies (Figure 1) have examined whether complement inhibition may slow GA growth. In this article, we review clinical trials for complement inhibitor therapy in GA over the past decade (completed: Table 1; ongoing: Table 2) and carefully assess the analytic approaches used in these trials and their limitations. Critical interpretation of the results allows for a better understanding of the role of complement inhibitor therapy in GA secondary to AMD, and can help guide future studies.

## 2. Methods

The search terms “AMD”, “GA” and “complement” were used to search clinical trials evaluating the safety and efficacy of complement inhibitors in GA in clinicaltrials.gov. All trials that included GA patients and in which the therapeutic intervention targeted a part of the complement pathway were included. For each drug, the website of the manufacturing pharmaceutical company was searched for information on clinical trials not included in clinicaltrials.gov.

## 3. Results

### 3.1. C3 Inhibition

The three pathways that lead to complement activation converge on the formation of a complement component 3 (C3) convertase (C3bBb or C4bC2b), which cleaves C3 to form C3a, an anaphylatoxin, and C3b, an active opsonin and, thus, triggers the common final pathway. Pegcetacoplan (APL-2, Apellis Pharmaceuticals, Inc., Waltham, MA, USA) is a synthetic cyclic peptide conjugated to a polyethylene glycol (PEG) polymer that binds to C3 and C3b, blocking C3 cleavage and preventing opsonization of targets for phagocytosis.

A phase 1b trial in 12 subjects with bilateral GA showed that patients tolerated pegcetacoplan at a dose of 15 mg/0.1 mL administered via intravitreal injection (NCT03777332). A multicenter, randomized phase 2 trial (the FILLY study; NCT02503332) was then carried out, enrolling 246 patients with GA secondary to AMD who were randomized in a 2:2:1:1 ratio to the following arms: a single 15 mg/0.1 mL dose monthly, a 15 mg/0.1 mL dose every other month (EOM), sham monthly and sham EOM. At 12 months, patients treated with monthly injections had a 29% reduction in the mean growth rate of GA lesions compared to those receiving sham injections (*p* = 0.008; 95% confidence interval: 9–49%) and those treated with EOM injections had a 20% reduction compared to the sham group (*p* = 0.067; 95% CI: 0–40%) [11]. This effect was more pronounced in the last six months of treatment, with rate reductions of 45% (*p* = 0.0004; 95% CI: 21–69%) in the monthly-treated group and 33% (*p* = 0.009; 95% CI: 9–58%) in the EOM-treated group. Overall, 47 prespecified genetic variants associated with AMD risk were not associated with GA lesion growth rate or response to treatment. In a post hoc analysis, there was a persistent effect of pegcetacoplan on GA growth even after controlling for known risk factors of GA in AMD, extrafoveal lesions and larger low luminance deficits [12]. In summary, the FILLY trial found that treatment of GA patients with intravitreal injections of pegcetacoplan was associated with a reduction in the rate of GA lesion growth, even after controlling for confounding factors.

**Figure 2:**
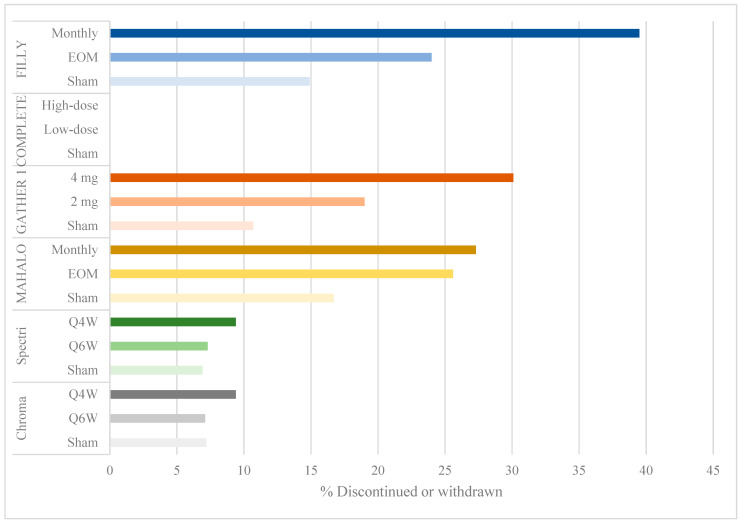
Bar graph showing percentage of discontinuation or withdrawal by study group.

Despite meeting its primary efficacy endpoint, the FILLY study had a few limitations. There was a withdrawal or discontinuation rate of 40% in the monthly group and 24% in the EOM group compared to 15% in the sham groups (Figure 2). A modified intention-to-treat analysis was done at 12 months including eyes that had received at least one injection and one examination. Given that up to 16% of eyes were lost to follow-up before month 12, the measurements included in the analysis were of eyes that responded well to treatment, or early measurements in patients that were later lost to follow-up. These limited measurements of GA lesions may have biased the results in favor of pegcetacoplan efficacy.

**Figure 3:**
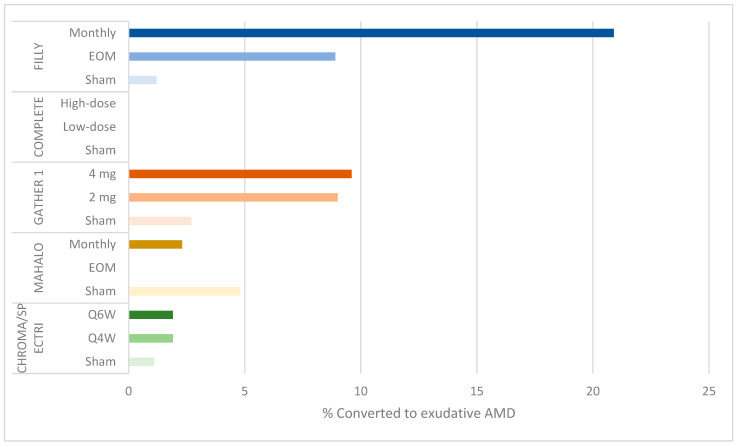
Bar graph showing percentage of conversion to exudative AMD by study group.

Additionally, the rates of serious ocular adverse events and conversion to eAMD resulting in withdrawal or discontinuation were dose related: 4.7% of subjects in the monthly group developed serious ocular adverse events (including endophthlamitis and elevated intraocular pressure) compared to 2.5% in the EOM group and 1.2% in the sham groups (Figure 2). Additionally, 20.9% (95% CI: 12.9–31%) of participants in the monthly group converted to eAMD, compared to 8.9% (95% CI: 3.6–17.4%) in the EOM group and 1.2% (95% CI: 0–6.7%) in the sham groups (Figure 3). While a post hoc analysis of FILLY data outlined some risk factors for conversion to eAMD, including baseline eAMD in the contralateral eye and a double-layer sign on ocular coherence tomography (OCT) in the study eye, there was a persistent dose-related association between complement inhibition and the development of eAMD, regardless of the presence of these risk factors [13].

Despite the limitations of the FILLY study, two phase 3 trials have been approved and are currently under way to evaluate the efficacy of pegcetacoplan in patients with GA (NCT03525613 and NCT03525600).

### 3.2. C5 Inhibition

In the terminal steps of the common final pathway of the complement cascade, complement component 5 (C5) is cleaved into C5a and C5b by C5 convertase (C4bC2bC3b or C3bBbC3b). C5a is an anaphylatoxin with a function similar to C3a, while C5b recruits other complement proteins (C6, C7, C8 and C9) to assemble the pathogen-killing MAC (C5b–C9). Antibodies and aptamers targeting C5 prevent its cleavage, and thus, prevent both recruitment of inflammatory cells and MAC formation. Given that C5 inhibition allows for residual upstream complement pathway activity, this strategy theoretically provides some benefits of complement inhibition while minimizing potential adverse effects.

Anti-C5 therapies being studied for treating GA include the antibodies eculizumab (Soliris^®^; Alexion Pharmaceuticals, Boston, MA, USA) and LFG316 (Novartis, Basel, Switzerland), as well as the aptamer avacincaptad pegol/ARC1905 (Zimura^®^; IVERIC Bio (formerly, Ophthotech), Cranbury, NJ, USA).

Eculizumab, which is a FDA-approved therapy for the treatment of paroxysmal nocturnal hemoglobinuria, underwent a randomized phase 2 trial for the treatment of GA secondary to non-exudative AMD between 2009 and 2013 (COMPLETE, NCT00935883). IV eculizumab administered for 24 weeks was well-tolerated by study subjects [14]. No participants were lost to follow-up by 26 weeks (Figure 2), and no serious adverse events were reported. At 26 and 52 weeks, there was no significant reduction in GA lesion size or drusen volume in eculizumab-treated eyes compared to the placebo group. Genetic analysis in the COMPLETE trial revealed that there was no association between the number of at-risk alleles at a given locus or the total number of alleles and the rate of GA lesion growth.

A second anti-C5 antibody therapy administered via intravitreal injection, LFG316, underwent a phase 1 trial, finding no serious adverse events at a dose of 5 mg (NCT01255462). A randomized, multicenter phase 2 trial comparing 5 mg monthly LFG316 injections with sham injections found no difference between the treatment and placebo groups in mean GA change from baseline or in visual acuity (NCT01527500) (Zamiri P. Complement C5 inhibition in AMD. Presentation at the Angiogenesis, Exudation and Degeneration meeting, 6 February 2016, Miami, FL, USA).

A third anti-C5 therapy is ARC1905, a pegylated RNA aptamer that binds and inhibits C5. A phase 1 trial showed tolerance to up to 2 mg administered via intravitreal injection (NCT00950638). A phase 2/3 trial (GATHER 1, NCT02686658) randomized 77 participants to 2 mg, 1 mg and sham injections in a 1:1:1 ratio in part 1, and 209 participants to 2 mg, 4 mg and sham injections in a 1:2:2 ratio in part 2, with treatment taking place over 12 months. Recently published results showed a 27.4% reduction in mean GA growth rate in the ARC1905 2 mg cohort compared to sham cohorts (*p* = 0.0072; 95% CI: 9.9–44.9%) and a 27.8% reduction in the 4 mg cohort compared to sham cohorts (*p* = 0.0051; 95% CI: 10.8–44.8%) [15].

Several limitations of this trial are similar to those in the FILLY trial of the pegylated C3 inhibitor, pegcetacoplan. There were high discontinuation and withdrawal rates, up to 30.1% in the 4 mg treatment arm, compared to 19% in the 2 mg arm and 10.7% in the sham group (Figure 2). Primary efficacy was evaluated using an intention-to-treat analysis including participants who received at least one dose of ARC1905. The authors implemented several sensitivity analyses to determine the validity of their findings. They used a mixed model for repeated measures (MMRM) to compare treatment groups, an approach that is valid under the assumption of missingness at random. However, given the high rate of withdrawal and discontinuation in treatment arms owing to adverse events and conversion to eAMD, the assumption of missingness at random is difficult to make. Subsequently, the authors conducted a sensitivity analysis using a fixed-value imputation (FVI) approach [16], concluding that treatment effects remained statistically significant. However, a FVI approach is limited by its inability to develop appropriate standard errors of intervention differences, resulting in variances that are too large or too small [16], thereby reducing the statistical validity of their efficacy results. Finally, the authors also carried out a “pattern-mixture-model imputation” approach evaluating the impact of missingness not at random, but they did not report whether their findings remained statistically significant. The high rates of missing data, therefore, remain a concern in evaluating the efficacy of ARC1905 in GA.

Another limitation is the high, dose-related rate of conversion to eAMD, which was also found in the FILLY study. At 12 months, 9.6% of participants in the 4 mg cohort and 9% in the 2 mg cohort developed choroidal neovascular membrane compared to 2.7% in the sham cohorts (Figure 3). These rates were higher at 18 months, 6 months after termination of the treatment: 15.7% in the 4 mg cohort and 11.9% in the 2 mg cohort compared to 2.7% in the sham cohort [17].

A separate phase 3 trial (GATHER 2, NCT04435366) evaluating the safety and efficacy of ARC1905 in patients with GA secondary to AMD is currently underway.

### 3.3. Factor D Inhibition

Factor D is a complement factor that activates C3 convertase in the alternative pathway. Lampalizumab (Genentech, San Francisco, CA, USA) is a monoclonal antibody therapy targeting factor D, administered by intravitreal injection. After a phase 1 trial showing a tolerance of lampalizumab up to a dose of 10 mg, a randomized, a multicenter phase 2 trial (the MAHALO study, NCT02288559) enrolled 123 subjects with GA secondary to AMD. Subjects were randomized in a 1:1:1 ratio to the following arms: 10 mg monthly injections, 10 mg EOM injections and sham injections [18]. At 18 months, there was a 20% reduction in lesion area progression among monthly-treated eyes compared to sham controls (*p* = 0.117; 80% CI 4 to 37%). Among subjects carrying the complement factor I (CFI) risk allele for AMD, the lesion area was reduced by 44% compared to sham controls (*p* = 0.0037). As in the FILLY and GATHER 1 trials, this phase 2 study also suffered from a high withdrawal rate from the treatment arm.

Positive results from the MAHALO study led to the initiation of two identical multicenter, randomized phase 3 trials, SPECTRI (NCT02247531) and CHROMA (NCT02247479). Each trial enrolled >900 subjects and compared intravitreal injection of lampalizumab every four or six weeks with sham injections. While lampalizumab was found to have a good safety profile at 48 weeks, it did not reduce GA lesion progression compared to sham injections in either trial [19]. Unlike findings from the MAHALO study, no benefit of lampalizumab was found among subjects with the CFI risk allele versus sham [19]. Given the lack of efficacy found at 48 weeks, both trials, originally intended for a duration of 96 weeks, were terminated early.

A few limitations in the MAHALO study may have resulted in the failure to meet primary efficacy endpoints in the subsequent phase 3 trials. There were high rates of discontinuation and withdrawal: 25.6% and 27.3% in the monthly and EOM treatment arms, respectively, compared to 16.7% in the sham group (Figure 2). The authors conducted a modified intention-to-treat analysis, including all patients with at least one treatment dose and one post-baseline fundus autofluorescence measurement. The last observation carried forward method was used to account for missing data, in which the last observation is carried forward in a participant with missing data. With more missing data in the treatment than in the control arms, the use of this carry forward method likely biased the results in favor of lampalizumab, showing less growth in GA lesion size. Unlike the C3 and C5 inhibitor trials, there were low rates of exudation or neovascular AMD. Furthermore, the authors used a pre-specified significance level of *p* < 0.2, thereby lowering their threshold for significant results.

### 3.4. Discontinuation Rates and Adverse Events in the FILLY, GATHER 1 and MAHALO Trials

Three of the trials reporting positive findings (FILLY, GATHER 1 and MAHALO) had discontinuation rates ranging from 19% to 39.5% in treatment arms compared to 10.7% to 16.7% in sham groups (Figure 2). The interpretation of the efficacy endpoint was made difficult by missing data from a large proportion of participants, as has been discussed in the limitations of each trial.

Reported reasons for discontinuation included patient withdrawal, sponsor or investigator decision, adverse events, a therapeutic alternative to the study drug, death unrelated to treatment and “other” (Table 3). Additionally, eyes that converted to eAMD were discontinued due to the potential impact on GA lesion size interpretation. The authors of the three trials did not report which adverse events led to dropout, or the reasons for patient, investigator and sponsor decisions to discontinue participation. It is therefore difficult to discern specific reasons for discontinuation of study participants. However, given the safety and efficacy data reported, two likely explanations for the high dropout rates are the high rates of serious and treatment-emergent adverse events (SAE and TEAE, respectively), as well as the high rates of conversion to eAMD, particularly in the FILLY and GATHER 1 trials.

Table 4 reports the rates of SAEs and TEAEs in the FILLY, GATHER 1 and MAHALO trials. In the FILLY study, up to 19.8% of treated patients were discontinued due to an adverse event, with rates of SAE reaching 4.7% in the monthly treatment arm. Of those, 2.3% were cases of endophthalmitis. In the MAHALO trial, 7–9.1% were discontinued due to an adverse event, including 2.4–6.8% of patients with an ocular SAE in the study eye. Specific events considered to be SAE in the MAHALO trial were not reported in the publication of the study’s results and are not available on clinicaltrials.gov. In the GATHER 1 trial, adverse events were only cited as a cause of discontinuation in 2.4% of patients receiving the 2 mg dose. While there were no SAEs reported in the study eyes of the GATHER 1 trial, the rates of TEAEs were 68.7% and 52.2% in the 4 mg and 2 mg groups, respectively, compared to 34.5% in the sham group. These adverse events may have contributed to the high rates of patient withdrawal (up to 15.7% in the 4 mg group).

### 3.5. Complement Inhibition and Increased Risk of Exudative AMD

In the FILLY study of the C3 inhibitor pegcetacoplan and the GATHER 1 study of the C5 inhibitor ARC1905, complement inhibition had the unintended effect of increasing the risk of neovascularization. No such finding was reported in the MAHALO trial of the factor 4 inhibitor lampalizumab, possibly due to variation in eligibility criteria [18,20]: the MAHALO trial excluded participants with contralateral eye eAMD. Indeed, a post hoc analysis of the FILLY study shows that baseline contralateral eAMD and study eye macular neovascularization may be risk factors for the onset of eAMD after complement inhibition. However, in eyes without these risk factors, there was a persistent, albeit lower, risk of eAMD in the treatment groups.

One possible explanation for the association between complement inhibition, risk of eAMD and GA lesion growth is that neovascularization limits the growth of GA lesions or confounds the interpretation of GA lesion size, thereby mediating the effect of complement inhibition on GA lesion growth. However, a repeat efficacy analysis in the FILLY study showed a persistent effect of complement inhibition on the rate of GA lesion growth when patients with eAMD were excluded, suggested that the reduction in GA lesion growth occurred independently of exudation.

Another possibility proposed by Keenan [20] in a response to the publication of GATHER 1 trial results is the assumption that there is a real effect of complement inhibition on GA lesion growth or incidence, and that the reduction of GA lesion size may subsequently increase the risk of eAMD. A final possibility is that complement inhibition may be driving two separate, unrelated processes: reduced GA lesion growth and increased neovascularization. Without a better understanding of the role of complement inhibition in these processes, the unintended effect of increased neovascularization after complement inhibition remains unexplained. Further investigation is needed to understand the complex role of complement in AMD pathogenesis.

### 3.6. Recent Targets of Complement Inhibition Therapy

#### 3.6.1. Recombinant Factor H

CFH negatively regulates the alternative pathway of complement activation. Since CFH variants are associated with increased risk of AMD, the Phase 2a ReGAtta study (NCT04643886) is being conducted to investigate whether intravitreal delivery of GEM103 (Gemini Therapeutics, Cambridge, MA, USA), a full-length recombinant CFH protein, is safe and may slow progression of non-exudative AMD in patients with loss-of-function CFH gene variants.

#### 3.6.2. Anti-Sense Oligonucleotides (ASO) and Gene Therapy

While complement inhibition strategies for GA that function at the protein level have existed for over a decade, newer therapies that target the gene expression level have recently emerged. IONIS-FB-LRx (Ionis Pharmaceuticals, Carlsbad, CA, USA) is an anti-sense oligonucleotide (ASO) that targets the gene encoding for complement factor B (CFB), which is involved in the alternative pathway of complement activation. IONIS-FB-LRx was recently shown to be safe when delivered subcutaneously and led to significant reduction in circulating CFB in healthy volunteers [21]. The Phase 2 GOLDEN study (NCT03815825) to assess the efficacy of IONIS-FB-LRx in slowing GA lesion growth is currently under way.

GT005 (Gyroscope Therapeutics, San Francisco, CA, USA) is an adeno-associated viral (AAV) vector encoding for CFI, a complement pathway inhibitor. Subretinal delivery of GT005 is safe and well-tolerated by mice and non-human primates and leads to durable CFI secretion in the eye [22]. The Phase 1/2 FocuS (NCT03846193), the Phase 2 EXPLORE (NCT04437368) and the Phase 2 HORIZON (NCT04566445) studies are currently under way to evaluate the safety and efficacy of GT005 for patients with GA. Finally, AAVCAGsCD59 (Hemera Biosciences, Waltham, MA, USA) is an AAV vector encoding for a soluble form of human CD59, which inhibits MAC formation and has been shown to attenuate CNV in a mouse model of AMD [23]. The Phase 1 HMR-1001 study (NCT03144999) has been completed, but the results have not yet been published.

## 4. Conclusions

In this review, we outline the results of clinical trials evaluating the safety and efficacy of complement inhibitors in eyes with GA secondary to AMD. The development of complement inhibitors for the treatment of GA was based on several genetic studies implicating complement dysregulation in AMD pathogenesis [2,3,4,5,6,7]. While risk alleles have been associated with susceptibility to geographic atrophy, they have not been associated disease progression [8,9]. Given the absence of a genetic association between complement inhibitors and GA disease progression, it is unsurprising that some complement inhibitor trials failed to show a reduction in GA lesion growth after complement inhibition. In studies with positive findings, high rates of withdrawal or discontinuation, as well as the increased dose-related risk of eAMD, are concerning and deserve further scrutiny. Additionally, the investigation of alternative components of the complement pathway and other stages of AMD (i.e., early to intermediate), may reveal more beneficial applications of complement inhibition and AMD progression, though significant work needs to be done to prove safety and clinical efficacy, which are both meaningful to the patients.

## Figures and Tables

**Table 1:** Completed studies designed to evaluate complement modulation as a novel therapeutic strategy for geographic atrophy (GA); results are current as of March 2021 based on reviewing clinicaltrials.gov.

Target	Drug	Study	Phase	N	Primary Outcome	% Dropout	Results
C3	Intravitreal pegcetacoplan	FILLY (NCT02503332)	2	246	GA growth (12 months)	Sham: 14.9% EOM: 24% Monthly: 39.5%	Monthly APL-2: 29% (95% CI: 10.2–47.8%) reduction in growth (*p* = 0.008); Every-other-month: 20% (95% CI: 0.5–39.5%) reduction (*p* = 0.067)
C5	Intravenous eculizumab (Soliris^®^; Alexion), anti-C5 monoclonal antibody	COMPLETE (NCT00935883)	2	60	GA growth and decrease in drusen volume (6 months)	Sham: 0 Low dose: 0 High dose: 0	No significant differences
	Intravitreal LFG316 (Novartis), anti-C5 monoclonal antibody	NCT01255462	1	24	Safety (85 days)	N/A	No safety or tolerability concerns
		NCT01527500	2	158	GA growth (505 days)	N/A	No significant difference
	Intravitreal avacincaptad pegol/ARC1905 (Zimura^®^; IVERIC Bio), anti-C5 aptamer	NCT00950638	1	47	Safety (1 year)	N/A	No safety or tolerability concerns
		GATHER1 (NCT02686658)	2/3	286	GA growth (12 months)	Sham: 10.7% 2 mg: 19% 4 mg: 30.1%	ARC1905 2 mg: 27.4% (95% CI: 9.9–44.9%) reduction in growth (*p* = 0.0072); 4 mg: 27.8% (95% CI: 10.8–44.8%) reduction (*p* = 0.0051)
CFD	Intravitreal lampalizumab (Genentech), anti-CFD antibody fragment	MAHALO (NCT01229215)	1b/2	143	GA growth (18 months)	Sham: 16.7% EOM: 25.6% Monthly: 27.3%	20% (80% CI: 4–37%) reduction in growth (*p* = 0.117)
		SPECTRI (NCT02247531)	3	975	GA growth (48 weeks)	Sham: 7.2% Q4W: 9.4% Q6W: 7.1%	No significant difference
		CHROMA (NCT02247479)	3	906	GA growth (48 weeks)	Sham: 6.9% Q4W: 9.4% Q6W: 7.3%	No significant difference

**Table 2:** Ongoing studies designed to evaluate complement modulation as a novel therapeutic strategy for geographic atrophy (GA); information is current as of March 2021 based on reviewing clinicaltrials.gov. * denotes anticipated enrollment.

Target	Drug	Study	Phase	N	Primary Outcome	Status
C3	Intravitreal pegcetacoplan	NCT03777332	1b	12 *	Safety (24 months)	Active, not recruiting
		DERBY (NCT03525613)	3	600 *	GA growth (12 months)	Active, not recruiting
		OAKS (NCT03525600)	3	600 *	GA growth (12 months)	Active, not recruiting
C5	Intravitreal avacincaptad pegol/ARC1905 (Zimura^®^; IVERIC Bio), anti-C5 aptamer	GATHER2 (NCT04435366)	3	400 *	GA growth (12 months)	Recruiting
CFH	Intravitreal GEM103 (Gemini), human CFH	ReGAtta (NCT04643886)	2a	60 *	Safety (18 months)	Recruiting
CFB	Subcutaneous IONIS-FB-LRx (Ionis), anti-sense inhibitor of CFB	GOLDEN (NCT03815825)	2	330 *	GA growth (49 weeks)	Recruiting
CFI	Subretinal GT005 (Gyroscope), adeno-associated viral (AAV) vector against CFI	FocuS (NCT03846193)	2	45 *	Safety (48 weeks)	Recruiting
		EXPLORE (NCT04437368)	2	75 *	GA growth (48 weeks)	Recruiting
		HORIZON (NCT04566445)	2	180 *	GA growth (48 weeks)	Recruiting
MAC	Intravitreal AAVCAGsCD59 (Hemera), AAV vector against sCD59	HMR-1001 (NCT03144999)	1	17	Safety (26 weeks)	Completed, results not published

**Table 3:** Reasons for discontinuation in the FILLY, GATHER 1 and MAHALO studies.

	FILLY			GATHER 1			MAHALO		
	Sham (*n* = 81)	EOM (*n* = 79)	Monthly (*n* = 86)	Sham (*n* = 84)	2 mg (*n* = 42)	4 mg (*n* = 83)	Sham (*n* = 42)	EOM (*n* = 44)	Monthly (*n* = 43)
Total discontinued	12 (14.8)	19 (24.1)	34 (39.5)	9 (10.7)	8 (19.0)	25 (30.1)	7 (16.7)	12 (27.3)	11 (25.6)
Patient withdrew, no. (%)	3 (3.7)	5 (6.3)	6 (7.0)	5 (6.0)	3 (7.1)	13 (15.7)	2 (4.8)	5 (11.4)	5 (11.6)
Sponsor/physician decision, no. (%)	1 (1.2)	1 (1.3)	6 (7.0)	3 (3.6)	4 (9.5)	10 (12.0)	3 (7.1)	3 (6.8)	2 (4.7)
Therapeutic alternative, no. (%)	0	0	0	0	1 (2.4)	0	1 (2.4)	0	1 (2.3)
Adverse event, no. (%)	4 (4.9)	5 (6.3)	17 (19.8)	0	0	1 (1.2)	2 (4.8)	4 (9.1)	3 (7.0)
Death unrelated to treatment, no. (%)	2 (2.5)	2 (2.5)	0	0	0	0	0	0	0
Other, no. (%)	2 (2.5)	6 (7.6)	5 (5.8)	1 (1.2)	0	1 (1.2)	0	0	0

**Table 4:** Rates of SAEs and TEAEs in the FILLY, GATHER 1 and MAHALO studies.

	FILLY			GATHER 1			MAHALO		
	Sham (*n* = 81)	EOM (*n* = 86)	Monthly (*n* = 79)	Sham (*n* = 84)	2 mg (*n* = 42)	4 mg (*n* = 83)	Sham (*n* = 42)	EOM (*n* = 44)	Monthly (*n* = 43)
Patients with at least one event, *n* (%)									
SAE in the study eye	1 (1.2)	2 (2.5)	4 (4.7)	0	0	0	1 (2.4)	3 (6.8)	0
Intraocular pressure increased	0	1 (1.3)	1 (1.2)	0	0	0	NR	NR	NR
Retinal detachment	0	0	1 (1.3)	0	0	0	NR	NR	NR
Endophthalmitis	0	1 (1.3)	2 (2.3)	0	0	0	NR	NR	NR
Intraocular inflammation	0	0	0	0	0	0	NR	NR	NR
TEAE in the study eye	47 (58)	49 (62)	65 (75.6)	38 (34.5)	35 (52.2)	57 (68.7)	24 (57.1)	30 (68.2)	36 (83.7)

NR = not reported.

## Data Availability

Not applicable.
